# The Relationship Between Lipid Profile as a Cardiovascular Risk Factor and Patient-Reported Physical Activity Scores: An Exploratory Analysis from the Saudi Systemic Lupus Erythematosus Cohort

**DOI:** 10.3390/jcm15124409

**Published:** 2026-06-07

**Authors:** Ibrahim Almaghlouth, Kawthar Bohliagah, Haya M. Almalag, Najma Khalil, Kazi Nur Asfina, Hebatallah Hamed Ali, Aos Aboabat, Fehaid Alanazi, Jiandong Su, Mohamed Bedaiwi, Mohammed A. Omair, Abdurhman S. Alarfaj

**Affiliations:** 1 Rheumatology Unit, Department of Medicine, College of Medicine, King Saud University, Riyadh 11451, Saudi Arabiambedaiwi@ksu.edu.sa (M.B.); asarfaj@ksu.edu.sa (A.S.A.); 2 Department of Clinical Pharmacy, College of Pharmacy, King Saud University, Riyadh 11451, Saudi Arabia; 3 College of Medicine Research Center, College of Medicine, King Saud University, Riyadh 11451, Saudi Arabia; 4 University Health Network, University of Toronto, Toronto, ON M5T 2S8, Canada

**Keywords:** systemic lupus erythematosus, dyslipidemia, cardiovascular risk, PROMIS, LupusQoL, physical function score, Saudi Arabia, patient-reported outcomes

## Abstract

**Background**: Systemic lupus erythematosus (SLE) is associated with an increased burden of cardiovascular disease (CVD), driven by dyslipidemia, hypertension, obesity, inflammation, and treatment. These factors can impact patient quality of life (QoL) by limiting physical activity. **Objectives**: To characterize lipid abnormalities as CVD risk factors in a Saudi SLE cohort and assess associations between lipid profile, SLE features, treatment, and patient-reported outcomes of physical activity. **Methods**: A cohort of adult SLE patients followed at King Saud University Medical City since 2021 was analyzed. Demographics, lipid profiles, blood pressure, BMI, SLEDAI-2K, SDI, disease duration, and treatment data were collected. Physical function and quality of life were assessed using the LupusQoL and PROMIS Physical Function T scores. Univariate and multivariate logistic regression analyses were conducted to identify associations between lipid abnormalities, SLE-related factors, and QoL physical activity measures. **Results**: A cohort of 169 patients (88.2% female, mean age 39.3 ± 12.4 years) was evaluated to assess the presence of dyslipidemia (23.7%), obesity (BMI ≥ 25, 66.3%), and hypertension (≥130/80 mmHg, 26.0%). Mean SLE duration was 9.2 ± 7.7 years and mean SLEDAI-2K was 11.0 ± 7.0. Among these patients, 52.7% used steroids, 88.2% used antimalarial drugs, and 53.8% used immunosuppressives. Dyslipidemia was associated with lower LupusQoL physical scores (adjusted OR 0.986; 95% CI 0.972–1.000; *p* = 0.0446). No significant associations were found between lipid levels and the PROMIS Physical Function T score. **Conclusions**: In this Saudi SLE cohort, dyslipidemia and other modifiable CVD risks were common. Dyslipidemia correlated with poorer LupusQoL-specific physical scores, which highlights the importance of lifestyle changes in patients with SLE.

## 1. Introduction

Patients with systemic lupus erythematosus (SLE) have a markedly elevated risk of atherosclerotic cardiovascular disease (CVD), attributable to both traditional risk factors, such as dyslipidemia, hypertension, and obesity, and disease-specific mechanisms, including chronic inflammation and long-term corticosteroid exposure [1,2,3,4,5,6]. Compared to the general population, SLE patients report lower health-related quality of life (HRQoL) [7]. Fatigue, depression, anxiety, pain, and low sleep quality are common in this population [8,9], and associated comorbidities, including dyslipidemia, hypertension, diabetes, obesity, and their complications, significantly affect patient outcomes and quality of life (QoL) [10,11,12,13]. SLE patients with a high cardiovascular risk profile have reported higher rates of depression and anxiety, with fatigue and sleep quality similar to other SLE populations [14].

In SLE, higher levels of moderate to vigorous physical activity and decreased sedentary time have been linked to lower blood pressure and reduced potential cardiovascular risk at 10 years [15]. Limited physical activity due to disease burden may increase cardiovascular risk, affecting QoL. Disease-specific quality of life should be assessed in patients with SLE due to the presence of CVD risk factors [16]. While quality of life has been well-studied in Western SLE cohorts, regional data from Saudi Arabia remain limited [11,12,13].

This study aimed to assess whether lipid abnormalities are a cardiovascular risk factor in a Saudi SLE cohort and the relationship with physical activity based on patient-reported outcomes using both a generic Patient-Reported Outcomes Measurement Information System (PROMIS) and a disease-specific lupus quality of life (LupusQoL) questionnaire.

## 2. Research Methodology

### 2.1. Study Design and Setting

This cross-sectional exploratory study assessed an ongoing cohort of adult SLE patients attending the rheumatology clinic at King Saud University Medical City (KSUMC) in Riyadh, Saudi Arabia. Data on this cohort has been collected since 2021 [17]. This study followed STrengthening the Reporting of OBservational studies in Epidemiology (STROBE) guidelines for observational cohort research [18]. Ethical approval was granted by the King Saud University Institutional Review Board (Protocol E-19-3955/58; 15 December 2019).

### 2.2. Participants

Adult patients (≥18 years) diagnosed with SLE by a rheumatologist with available lipid profiles and completed PROMIS and LupusQoL assessments were included. Patients without recent lipid data were excluded.

### 2.3. Variables, Data Sources, and Measurements

Dyslipidemia was defined as total cholesterol > 5.2 mmol/L, HDL < 1.0 mmol/L, or LDL > 3.4 mmol/L according to the National Cholesterol Education Program (NCEP) Adult Treatment Panel III (ATP III). Hypertension was defined using American College of Cardiology/American Heart Association (ACC/AHA ≥ 130/80 mmHg) and conventional (≥140/90 mmHg) thresholds. BMI ≥ 25 was classified as overweight/obesity. Disease activity and damage were evaluated using the Systemic Lupus Erythematosus Disease Activity Index 2000 (SLEDAI-2K) and the Systemic Lupus International Collaborating Clinics/American College of Rheumatology Damage Index (SLICC/ACR Damage Index (SDI)) [19,20]. Medication data included steroid, antimalarial, and immunosuppressive use.

Patient-reported outcomes included the PROMIS Physical Function T score and the LupusQoL physical domain [11,12,13]. The outcome measures in the PROMIS instrument were the PROMIS scores for fatigue, physical functioning, pain, and social interaction. These were measured using the questions from the 29-item PROMIS profile version 2.1 assessing these four domains [11], which includes 8 items pertaining to fatigue, 10 items pertaining to physical functioning, 3 items pertaining to pain intensity, and 3 items pertaining to social interaction. Questions about pain were scored on a 10-point Likert scale, while other domains were scored on a 5-point Likert scale. For each domain, item scores were summed to create four separate composite scores, and each composite score was transformed into a T score for each patient using the PROMIS metric system website. The score was calibrated with a US average score of 50 and a standard deviation of 10 as a reference group. The T score was then categorized according to PROMIS standardized cut-off points on the health measures website as normal, mild, moderate, and severe for fatigue, pain, and physical functioning, while social interaction was categorized as very high, high, average, low, and very low.

The LupusQoL physical score instrument consists of 34 disease-specific questions across 8 domains [12]. Specifically, questions 1 to 8 measure physical health, questions 9 to 14 measure emotional health, questions 15 to 19 measure body image, questions 20 to 22 measure pain, questions 22 to 25 assess planning, questions 26 to 29 measure fatigue, and questions 30 to 34 assess other aspects of quality of life. Each QoL question uses a 5-point Likert scale (0 = all of the time, 1 = most of the time, 2 = a good bit of the time, 3 = occasionally, and 4 = never). Item response scores are added up for each domain, and the mean raw domain score is obtained by dividing the total score by the number of items in that domain. The mean raw domain score is converted to scores ranging from 0 (worst HRQoL) to 100 (best HRQoL) by dividing by 4 (the 5 Likert responses minus 1) and then multiplying by 100 (mean raw domain score ÷ 4) × 100 = transformed score for the domain) [12]. These questionnaires were administered at baseline and follow-up visits [17].

### 2.4. Statistical Analysis

The SAS v.9.4 (SAS Institute)software package was used for analyses.. Continuous variables are expressed as mean ± SD, and categorical variables are expressed as percentages. Group differences were initially assessed using t-tests and chi-square tests. Logistic regression was used to identify predictors of dyslipidemia, and linear models were used to examine associations between lipid measures and physical activity. Multivariable regression models adjusted for demographics (age and gender), disease characteristics, and treatment were used. Significance was set at *p* < 0.05.

## 3. Results

### 3.1. Demographics and Descriptives

A total of 169 patients were included after excluding 120 patients without available lipid data. The cohort was 88.2% female, with a mean age of 39.3 ± 12.4 years. Dyslipidemia was prevalent in almost a quarter of SLE patients (23.7%), overweight/obesity was observed in 66.3%, and hypertension (ACC/AHA ≥ 130/80 mmHg) was identified in 26.0%. Diabetes was present in 8.9% of patients, while statin use was low (4.7%). Most participants were non-smokers (74.0%). Mean total cholesterol, HDL, and LDL were 4.6 ± 1.1, 1.5 ± 0.5, and 2.6 ± 0.8 mmol/L, respectively (Table 1).

Mean disease duration was 9.2 ± 7.7 years, with moderate activity (mean SLEDAI-2K 11.0 ± 7.0; mean SDI 0.5± 0.8). Over half of patients were using steroids, nearly nine in ten were using antimalarials, and over half were using immunosuppressive agents. Physical function was assessed using the LupusQoL physical score (mean 73.6 ± 25.0) and the PROMIS Physical Function T score (mean 45.3 ± 8.5) (Table 2).

### 3.2. Inferential Statistics and Regression Models

Logistic regression analysis revealed that dyslipidemia was significantly associated with a lower LupusQoL physical score (OR = 0.983, 95% CI [0.970–0.996], *p* = 0.0128). No significant associations were found between lipid parameters and PROMIS Physical Function T scores (Table 3).

SLE duration was associated with dyslipidemia risk (OR = 1.055, *p* = 0.0214), although this was borderline in the multivariate logistic regression analysis (OR = 1.046, 95% CI [0.998–1.097], *p* = 0.0587).

Multivariate logistic regression confirmed the association between dyslipidemia and lower LupusQoL physical score (OR = 0.986, 95% CI [0.972–1.000], *p* = 0.0446). Other variables, including age, gender, disease activity, and treatment, were not significant predictors of dyslipidemia (Table 4).

## 4. Discussion

This study shows a substantial burden of modifiable cardiovascular risk factors in Saudi patients with SLE. The prevalence of dyslipidemia (23.7%), overweight/obesity (66.3%), and hypertension (26%) highlights the need for proactive screening and intervention. The independent association between dyslipidemia and reduced lupus-specific QoL underscores the interplay between metabolic health and patient well-being. CVD has a significant impact on many aspects of SLE patients’ lives and their overall HRQoL, including improved survival and longer disease duration [5,21,22]. In addition to the burden of the disease itself, people with SLE must also navigate treatment and comorbidities, further affecting their HRQoL [23]. HRQoL measurement using LupusQoL and PROMIS provides an opportunity for SLE patients to participate in disease management and may facilitate better communication with health professionals [24].

Generic patient-reported outcome (PRO) tools and lupus-specific tools have been employed to assess health-related quality of life in SLE patients, including the Medical Outcomes Study Short Form 36 (SF-36) and LupusQoL in UK, Canadian, and French studies [25,26,27]. Studies reporting on HRQoL usually employ the SF-36, while LupusQoL is a newly developed disease-specific HRQoL. These tools assess the physical, psychological, and social effects of the disease on SLE patients. Most studies have employed the SF-36 to assess HRQoL [12,25]. Here, while the physical domain was not affected in the LupusQoL, differences were found using the SF-36. The National Institutes of Health Initiative Patient-Related Outcome Measurement Information System (PROMIS) was established to support self-reporting across a range of domains, including fatigue and pain [11]. PROMIS-29 is suitable for assessing quality of life in SLE patients to identify deficits in domains including fatigue, pain, and physical function, with pain being a significant independent contributor to poorer HRQoL. SLE patients score worse than the general population across most domains of the PROMIS-29 scale [28,29].

In English-speaking Asian patients with SLE, mean PROMIS T scores for fatigue, pain interference, anxiety, sleep disturbance, sleep-related impairment, and physical function were worse than those in the general U.S. population [30,31]. In our study, no significant associations between lipid parameters (total cholesterol, HDL, LDL) and PROMIS Physical Function T scores were found, indicating that while dyslipidemia is prevalent, it does not directly impair self-reported physical function in this population.

Studies have shown that different manifestations of SLE affect QoL across domains [31,32,33]. For instance, one study has shown that HRQoL is significantly compromised in obese SLE patients [34]. In contrast, some studies have found no relationship between patients’ age, disease duration, and quality of life, while others report that age and disease duration affect QoL in SLE [24,33]. In our study, disease duration, but not age, was a predictor of poor QoL. Disease severity has also been associated with worse symptoms and poorer HRQoL [28].

Other clinical characteristics, including disease activity measured through SLEDAI and SDI scores, affect various domains of QoL, including pain, fatigue, and sleep quality [24,35]. Compared to healthy subjects, SLE patients have a poorer QoL when the disease is inactive and in association with worsened disease severity [24,28,36]. Disease and organ damage are also predictors of poor QoL [24,35]. However, some studies have reported that worse SLEDAI-2K scores do not affect QoL, while QoL may be affected by other disease-related factors [32]. In this study, the borderline significant association between dyslipidemia and disease duration suggests that the cumulative impact of SLE over time may increase cardiovascular risk factors. This implies that cardiovascular risk in this cohort is more closely related to SLE-specific factors, particularly disease duration. These findings align with the literature linking prolonged disease duration and glucocorticoid exposure to adverse lipid profiles [5,22].

Lower QoL in SLE patients has been correlated with various SLE manifestations, including cutaneous, neuropsychiatric, and musculoskeletal conditions and lupus nephritis [32,36,37,38,39,40], while some studies have reported contrasting findings regarding cutaneous manifestations and quality of life [37]. Multivariate logistic regression confirmed the association between dyslipidemia and LupusQoL physical score in our cohort, implying that dyslipidemia could independently contribute to reduced physical quality of life in SLE. The lack of association between patients’ lipid profile and PROMIS score may be explained by the fact that LupusQoL may be more sensitive to subtle lupus-related impairments, while PROMIS may not fully capture lupus-specific burdens.

Based on the present findings and previous reports, unlike generic PROMIS Physical Function scores, the LupusQoL physical domain may better capture disease-specific limitations affected by lipid abnormalities [11,12,13]. This study contributes to the literature by demonstrating the link between lipid profile disturbances and physical activity levels and their potential role in increasing cardiovascular risk in patients with SLE. However, limitations include the single-center design, limited statin use, and missing education/smoking data, as well as the possibility of selection bias after excluding patients with missing data, which may restrict generalizability. Nevertheless, this study is one of the few to characterize CVD risk and QoL in a Middle Eastern SLE population.

The high prevalence of obesity, hypertension, and dyslipidemia in this population underscores the need for comprehensive cardiovascular risk management. Regular lipid profile monitoring, alongside lifestyle interventions and targeted treatment, may reduce cardiovascular risk and improve SLE patients’ quality of life.

## 5. Conclusions

Among Saudi adults with SLE, dyslipidemia and other modifiable cardiovascular risk factors are common. Dyslipidemia is independently associated with poorer lupus-specific physical quality of life, emphasizing the need for integrated cardiometabolic monitoring and prevention strategies in SLE care.

## Figures and Tables

**Table 1:** Demographics and cardiovascular characteristics in SLE patients.

Characteristic	Mean ± SD/No. (%)
Age (years)	39.3 ± 12.4
Female	149 (88.2%)
Obesity (BMI ≥ 25)	112 (66.3%)
Non-smoker	125 (74.0%)
Total cholesterol (mmol/L)	4.6 ± 1.1
HDL (mmol/L)	1.5 ± 0.5
LDL (mmol/L)	2.6 ± 0.8
Dyslipidemia	40 (23.7%)
Diabetes	15 (8.9%)
Hypertension ≥ 130/80 mmHg	44 (26.0%)
Hypertension ≥ 140/90 mmHg	31 (18.3%)
Statin therapy	8 (4.7%)

**Table 2:** SLE-related characteristics, treatment, and physical function scores (*n* = 169).

Characteristic	Mean ± SD/n (%)
SLE duration (years)	9.2 ± 7.7
SLEDAI-2K score	11.0 ± 7.0
SLICC/ACR Damage Index (SDI) score	0.5 ± 0.8
Steroid therapy	89 (52.7)
Antimalarial therapy	149 (88.2)
Immunosuppressive therapy	91 (53.8)
Lupus nephritis (biopsy-proven)	65 (38.5)
LupusQoL physical score	73.6 ± 25.0
PROMIS Physical Function T score	45.3 ± 8.5

**Table 3:** Univariate analysis of lipid profile and physical function tests with *p*-value of difference using lipid profile/dyslipidemia as dependent variables and physical function tests as independent variables.

Characteristic	LupusQoL Physical Score			PROMIS Physical Function T Score		
	Parameter Estimate	Standard Error	*p*-Value	Parameter Estimate	Standard Error	*p*-Value
Total cholesterol (mmol/L)	−0.004	0.003	0.3012	0.003	0.010	0.7633
HDL (mmol/L)	0.002	0.002	0.3763	0.009	0.005	0.0916
LDL (mmol/L)	−0.004	0.003	0.1481	−0.004	0.008	0.6579
	Odds Ratio	95% CI of OR	*p*-value	Odds Ratio	95% CI of OR	*p*-value
Dyslipidemia	0.983	(0.970–0.996)	0.0128 *	0.974	(0.933–1.017)	0.585

CI = confidence interval; * significant at *p* < 0.05; LupusQol = Lupus Erythematosus Quality of Life Questionnaire; PROMIS = Patient-Reported Outcomes Measurement Information System; HDL = high-density lipoprotein; LDL = low-density lipoprotein.

**Table 4:** Predictors of dyslipidemia based on logistic regression analysis.

Characteristic	Univariate OR (95% CI)	*p*-Value	Multivariable OR (95% CI)	*p*-Value
Age (years)	1.027 (0.998–1.057)	0.0701	-	-
Female	0.929 (0.315–2.738)	0.8941	-	-
SLE duration (years)	1.055 (1.008–1.104)	0.0214 *	1.046 (0.998–1.097)	0.0587
SLEDAI-2K	1.015 (0.966–1.066)	0.5576	-	-
SLICC SLICC/ACR Damage Index (SDI) score	1.369 (0.900–2.082)	0.142	-	-
Steroid therapy	1.113 (0.545–2.270)	0.769	-	-
Antimalarial therapy	0.640 (0.226–1.812)	0.4011	-	-
Immunosuppressive therapy	0.704 (0.345–1.434)	0.3334	-	-
LupusQoL physical score	0.983 (0.970–0.996)	0.0128 *	0.986 (0.972–1.000)	0.0446 *
PROMIS Physical Function T score	0.974 (0.933–1.017)	0.585	-	-

CI = confidence interval; * significant at *p* < 0.05; LupusQol = Lupus Erythematosus Quality of Life Questionnaire; HDL = high-density lipoprotein; LDL = low-density lipoprotein.

## Data Availability

All data generated or analyzed during this study are included in this published article. Further inquiries can be directed to the corresponding author.

## Abbreviations

SLE	Systemic lupus erythematosus
CVD	cardiovascular disease
HRQoL	health-related quality of life
QoL	quality of life
PROMIS	Patient-Reported Outcomes Measurement Information System
LupusQoL	lupus quality of life
STROBE	STrengthening the Reporting of OBservational studies in Epidemiology
SLEDAI-2K	Systemic Lupus Erythematosus Disease Activity Index 2000
SLICC/ACR Damage Index (SDI)	Systemic Lupus International Collaborating Clinics/American College of Rheumatology Damage Index
SDI	SLICC/ACR Damage Index
ACC/AHA	American College of Cardiology/American Heart Association
PRO	patient-reported outcome
